# Interaction Between Arteriosclerosis and Amyloid-β on Cognitive Function

**DOI:** 10.3233/JAD-230604

**Published:** 2024-01-16

**Authors:** Ingeborg Frentz, Joyce van Arendonk, Anna E. Leeuwis, Meike W. Vernooij, Wiesje M. van der Flier, Daniel Bos, Peter Paul De Deyn, Frank J. Wolters, M. Arfan Ikram

**Affiliations:** a Department of Epidemiology, Erasmus MC, Rotterdam, The Netherlands; b Department of Neurology, UMCG, Groningen, The Netherlands; c Department of Radiology & Nuclear Medicine, Erasmus MC, GD Rotterdam, The Netherlands; d Department of Neurology, Alzheimer Center Amsterdam, Amsterdam Neuroscience, Vrije Universiteit Amsterdam, Amsterdam UMC, Amsterdam, The Netherlands; e Department of Epidemiology, Vrije Universiteit Amsterdam, Amsterdam UMC, Amsterdam, The Netherlands; f Alzheimer Centre Groningen, UMCG, Groningen, The Netherlands

**Keywords:** Alzheimer’s disease, amyloid-β, arteriosclerosis, calcification, dementia, plasma biomarkers

## Abstract

**Background:**

Dementia is a multifactorial disease, with Alzheimer’s disease (AD) and vascular pathology often co-occurring in many individuals with dementia. Yet, the interplay between AD and vascular pathology in cognitive decline is largely undetermined.

**Objective:**

The aim of the present study was to examine the joint effect of arteriosclerosis and AD pathology on cognition in the general population without dementia.

**Methods:**

We determined the interaction between blood-based AD biomarkers and CT-defined arteriosclerosis on cognition in 2,229 dementia-free participants of the population-based Rotterdam Study (mean age: 68.9 years, 52% women) cross-sectionally.

**Results:**

Amyloid-β (Aβ)_42_ and arterial calcification were associated with cognitive performance. After further adjustment for confounders in a model that combined all biomarkers, only arterial calcification remained independently associated with cognition. There was a significant interaction between arterial calcification and Aβ_42_ and between arterial calcification and the ratio of Aβ_42/40_. Yet, estimates attenuated, and interactions were no longer statistically significant after adjustment for cardio metabolic risk factors.

**Conclusions:**

Arteriosclerosis and AD display additive interaction-effects on cognition in the general population, that are due in part to cardio metabolic risk factors. These findings suggest that joint assessment of arteriosclerosis and AD pathology is important for understanding of disease etiology in individuals with cognitive impairment.

## INTRODUCTION

Alzheimer’s disease (AD) and vascular pathology are the most common causes of dementia, and can been found jointly in about 40% of individuals with dementia at time of death [1, 2]. Arteriosclerosis is a generic term that encompasses the loss of arterial wall elasticity related to stiffening of vessels. While the prevalence of both arteriosclerosis and AD pathology steeply increases with age, individually neither amyloid deposition nor arteriosclerosis by itself are generally sufficient to cause the cognitive impairment [3–5], as many individuals with either of these pathologies never develop dementia. Against this backdrop, the co-occurrence of different pathologies is an important determinant of developing cognitive impairment and dementia [6–8], with potential implications for diagnosis as well as treatment indication.

In recent years, various plasma biomarkers have been developed into valid markers of central nervous system pathology. With the availability of these less expensive and less invasive plasma alternatives to cerebrospinal fluid and PET investigation, it becomes possible to study the interplay between different pathophysiological processes *in vivo* in adequately powered studies of unselected individuals. Plasma levels of amyloid-β (Aβ)_40_ and Aβ_42_ can identify AD pathology and risk of AD [9, 10], whereas brain magnetic resonance imaging (MRI) and computed tomography (CT) provide good markers for small-vessel disease and arteriosclerosis, respectively. Using non-contrast CT, we have recently shown in non-demented individuals that plasma Aβ_40_ is associated with arteriosclerosis [11], suggesting an interplay between these pathologies in the preclinical disease stage. This adds to earlier MRI studies showing that Aβ was associated with subclinical markers of vascular brain disease [12–14].

Aβ is considered a hallmark of AD, but it remains unclear what the exact cause and consequence of accumulation is [15], and while much evidence indicates that vascular pathology is a major risk factor for AD and dementia, it is unknown if vascular brain disease causes, amplifies or precedes development of AD [15]. Furthermore, the impact of a joint contribution of vascular and amyloid pathology on cognitive outcomes *in vivo* is largely undetermined [14].

The aim of the present study was to examine the joint effect of arteriosclerosis and AD pathology on cognition in the general population without dementia.

## METHODS

### Study population

This study is embedded in the Rotterdam Study, an ongoing population-based cohort study in the Netherlands, details of which have been described previously [16]. The original study population in 1990 consisted of 7,983 participants aged≥55 years from the Ommoord area, a suburb of Rotterdam. In 2000, the cohort was expanded with 3,011 persons who had reached age 55 years or had moved into the study area. Follow-up examinations take place at a dedicated research center every 4 years. Baseline of the current study was the 4^th^ examination round of the original cohort, and the 2^nd^ examination round of the expansion cohort. Between 2002 and 2005, 5,094 participants had blood samples taken during their visit to the study center. We excluded 518 participants with missing or invalid test results for plasma Aβ_40_ or plasma Aβ_42_. Of the remaining 4,576 participants, a subsample of 2,252 underwent on-contrast CT examination of the heart, aorta, and carotid arteries up till the level of the circle of Willis. Measures of arterial wall elasticity were not available in this examination round of the Rotterdam Study cohort. Of these participants, we excluded 23 with extreme values (exceeding±3.5 SD), the remaining sample consisted of 2,229 participants.

### Ethics statement

The Rotterdam Study has been approved by the Medical Ethics Committee of the Erasmus MC (registration number MEC 02.1015) and by the Dutch Ministry of Health, Welfare and Sport (Population Screening Act WBO, license number 1071272-159521-PG). The Rotterdam Study Personal Registration Data collection is filed with the Erasmus MC Data Protection Officer under registration number EMC1712001. The Rotterdam Study has been entered into the Netherlands National Trial Register (NTR; https://www.trialregister.nl) and into the WHO International Clinical Trials Registry Platform (ICTRP; https://apps.who.int/trialsearch/) under shared catalogue number NL6645/NTR6831. All participants provided written informed consent to participate in the study and to have their information obtained from treating physicians.

### Measurement of plasma biomarkers

EDTA plasma was sampled, aliquoted, and frozen at –80°C according to standard procedures. Measurements were carried out in two separate batches; the first batch included 2,000 samples, obtained from a random selection of 1,000 participants from the fourth visit of RS-I and 1,000 from the second visit of RS-II. The second batch included samples from all the remaining participants of these two study waves. All measurements were performed at Quanterix (Lexington, MA, USA) on a single molecule array (Simoa) HD-1 analyzer platform [17]. Detailed methods have been described previously [10].

### Quantification of arteriosclerosis

A 16-slice (*n* = 663) or 64-slice (*n* = 1,589) multidetector CT scanner (Somatom Sensation 16 or 64; Siemens, Forchheim, Germany) was used to perform non-contrast CT scanning. We obtained images of the coronary arteries, aortic arch, extracranial internal carotid arteries, and intracranial internal carotid arteries. Detailed information regarding imaging parameters has been described previously [18–20]. Calcification in the coronary arteries, aortic arch and the extracranial internal carotid arteries was quantified using commercially available software (SyngoCalciumScoring; Siemens). Calcification was measured in the left main, left anterior descending, left circumflex, and right coronary arteries. The aortic arch was measured from the origin to the first centimeter of the common carotid arteries, the vertebral arteries, and the subclavian arteries beyond the origin of the vertebral arteries. The extracranial carotid arteries were measured at both sides, within 3 centimeters proximal and distal of the bifurcation [18]. For the intracranial internal carotid arteries we used a separate, semi-automated scoring method to quantify calcification [21–23]. Briefly, after manually delineating calcification in the trajectory of the intracranial internal carotid artery, the calcification was calculated by multiplying the number of pixels above the threshold for calcium (i.e., 130 Hounsfield units) [24] with the pixel size and slice increment [19, 20]. Scoring of calcification was done semi-automatically by 3 trained raters, the interrater reliability was very good (intraclass correlation coefficient, 0.99) [21]. Examples of calcification in examined vessel beds can be seen in Supplementary Figure 1. Next, we used these calcification s from the four arteries to determine a single measure of the systemic burden of calcification using a summary metric for arterial calcification, the “C-factor” [25]. The C-factor is calculated using a principal component analysis including calcification levels in the coronary artery, aortic arch, extracranial carotid artery, and intracranial carotid artery. A more detailed description of the calculation method can be found elsewhere [25].

**Fig. 1:**
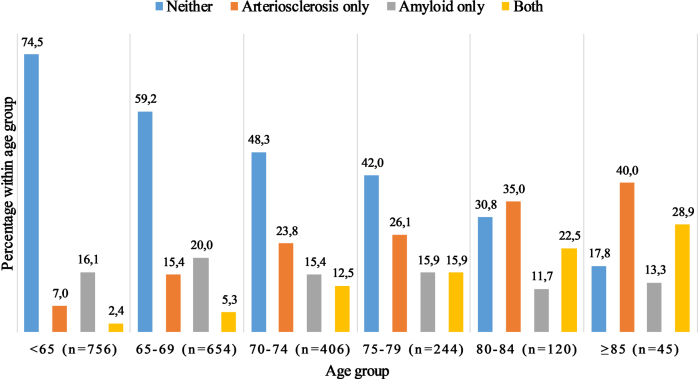
Co-occurrence of arteriosclerosis and amyloid pathology. The figure depicts the number of participants with neither pathology, arteriosclerosis only, amyloid pathology only or co-occurrence of both pathologies, stratified by age groups. Presence of arteriosclerosis was defined as the highest quartile of the C-factor, whereas presence of amyloid was defined as the lowest quartile of the Aβ_42/40_ ratio.

### Measures of cognition

Cognitive function was assessed with a neuropsychological test battery comprising the verbal fluency test, the letter-digit substitution task, a 15-word learning test (immediate and delayed recall), the Stroop test, and Purdue pegboard task [26]. For all participants, z-scores were calculated for each test separately by dividing the difference between the individual and mean test scores by the standard deviation. To obtain a measure of global cognitive function, we calculated a standardized compound score (g-factor) using principal component analysis [26]. We calculated scores for cognitive domains for memory (word learning test, immediate and delayed recall), executive function (Stroop interference task, verbal fluency test, and letter-digit substitution task [weighted half]), information processing (Stroop reading and color naming task and letter-digit substitution task [weighted half]), and motor function (Purdue pegboard test).

### Covariables

For each participant, we obtained information on highest level of completed education (classified into lower, intermediate, and higher education), history of smoking (i.e., current, former, or never), and alcohol consumption. Hypertension was defined as a blood pressure >140/90mmHg or the use of blood-pressure lowering medication for this indication. Body mass index was computed from measurements of height and weight (kg/m^2^). Blood samples were obtained at baseline to determine serum total cholesterol and glucose. Apolipoprotein E4 (*APOE* ɛ4) genotype was determined by polymerase chain reaction on coded DNA samples in the original cohort, and by biallelic Taqman assays (TaqMan Gene Expression Assays; Thermo Fisher Scientific, Waltham, Massachusetts) (rs7412 and rs429358) for the expansion cohort. *APOE* genotype was classified as *APOE* ɛ4 carrier (≥1 ɛ4 allele) or noncarrier.

### Statistical analysis

Because Aβ_42_ had a skewed distribution, we used natural log-transformed values. We subsequently standardized Aβ_40_, Aβ_42_, the Aβ_42/40_ ratio, and cognition measurements by dividing the difference between the individual value and the population mean by the population standard deviation. Missing data ranged from 0.1% for hypertension to 9.4% for creatinine level and were imputed 10 times with 20 iterations using chained equations (MICE R package). Distribution of covariables was similar in the imputed and non-imputed datasets.

We determined the association of arterial calcification, C-factor and per calcification tertiles (no calcification present and calcification present in tertiles), and plasma biomarkers with global cognition using linear regression models. We adjusted the association of the Aβ_40_ measurements for Aβ_42_, and Aβ_42_ for Aβ_40_ in all models. To adjust for confounding, we constructed two models. In the first model, we adjusted for age, age^2^, sex, batch number of the plasma analysis, scanner type used for CT, educational level and the time interval between scan acquisition and cognitive assessment. In the second model, we additionally adjusted for cholesterol, HDL, body mass index, smoking status, diabetes, lipid lowering medication, systolic blood pressure, diastolic blood pressure, blood pressure lowering medication, *APOE*ɛ4 carriership, creatinine level and alcohol consumption. In a final model, we assessed independent effects of Aβ and calcification by mutual adjustment for arterial calcification and the plasma biomarkers.

Finally, we assessed the interaction effects between the C-factor and plasma biomarkers on global cognition and cognitive domains by addition of an interaction term to the linear regression models and stratifying on the biomarker level. Plots for the biomarker levels were stratified on low, mean, and high tertiles of the biomarker level. All statistical analyses were performed in R version 4.0.3 (R Foundation for Statistical Computing, Vienna, Austria).

## RESULTS

Characteristics of the study population are shown in Table 1. The mean age was 68.9 years (SD 6.6), and 1,158 (52%) participants were women. Prevalence of calcification ranged from 73% in the extracranial carotid arteries to 93% in the aorta, both increasing steeply with age. The C-factor explained 66% of variation in calcification across the different arteries in this population. Severe calcification (highest quartile) was present in combination with a low plasma Aβ_42/40_-ratio (lowest quartile) in 183 (8.2%) of participants, and co-occurrence increased with age from 2.4% under the age of 65 to 28.9% after age 85 (Fig. 1). A correlation matrix of Aβ, calcification and all covariables is presented in Supplementary Figure 2. Levels of plasma Aβ_40_ and Aβ_42_ were highly correlated (*r* = 0.58). Aβ_40_ and the C-factor were positively correlated with age (Supplementary Fig. 2).

**Table 1:** Characteristics of the study population (N = 2,229), average over 10 imputed datasets

	Study population
	(N = 2,229)
Age (y)	68.9 (±6.6)
Female sex	1,158 (52.0%)
Education
Lower	695 (31.2%)
Intermediate	1,104 (49.5%)
Higher	430 (19.3%)
Smoking
Never	640 (28.7%)
Former	1,246 (55.9%)
Current	343 (15.4%)
Alcohol (grams/day; median, IQR)	9.3 (1.4–20.0)
Body mass index (kg/m^2^)	27.6 (±3.9)
Systolic blood pressure (mmHg)	146.5 (±20.1)
Diastolic blood pressure (mmHg)	80.3 (±10.7)
Blood pressure lowering medication	880 (39.5%)
Serum total cholesterol (mmol/L)	5.7 (±1.0)
Serum high-density lipoprotein (mmol/L)	1.4 (±0.4)
Lipid lowering medication	979 (43.9%)
Diabetes	230 (10.3%)
Creatinine level (μmol/L)	82.1 (±18.8)
*APOE* ɛ4 carriers	628 (28.2%)
Aβ_40_ (pg/mL; median, IQR)	247.9 (222.9–279.8)
Aβ_42_ (pg/mL; median, IQR)	10.1 (8.7–11.7)

SD, standard deviation; IQR, interquartile range. Data are presented as frequency (%) for categorical and mean±SD for continuous variables unless indicated otherwise.

### Amyloid-β, calcification, and cognition

Arterial calcification was associated with worse cognitive performance (β[95% CI] per standard deviation increase: –0.06 [–0.08; –0.04]), independent of cardiovascular risk factors and concurrent plasma levels of Aβ in multivariable analyses (–0.04 [–0.07; –0.01]) (Table 2). Plasma Aβ_42_ was associated with cognition in the age- and sex-adjusted model (β[95% CI]: 0.06 [0.01; 0.11]), but estimates attenuated and were no longer statistically significant in the further adjusted model (Table 2). Aβ_40_ and the ratio of Aβ_42/40_ were not significantly associated with cognitive performance (Table 2).

**Table 2:** Association of plasma biomarkers (Aβ_40_, Aβ_42_) and systemic arterial calcification (C-factor) on global cognition outcome (g-factor)

	Global cognition (g-factor)		
	Adjusted mean difference (95% Confidence Interval)		
	Model I	Model II	Model III
Aβ_40_	–0.04 (–0.09; 0.01)	–0.01 (–0.06; 0.04)	–0.01 (–0.05; 0.05)
Aβ_42_	0.06 (0.01; 0.11)^*^	0.02 (–0.04; 0.07)	0.01 (–0.04; 0.07)
Aβ_42/40_ ratio	0.03 (–0.01; 0.07)	0.001 (–0.05; 0.05)	0.001 (–0.05; 0.04)
Arterial calcification (C-factor)	–0.06 (–0.08; –0.04)^**^	–0.04 (–0.07; –0.01)^**^	–0.04 (–0.07; –0.01)^**^

^*^p < 0.05; ^**^p < 0.01. Model I: adjusted for age, age^2^, sex, education, batch number (for plasma analysis only), and scanner used and time interval between scanning and cognitive assessment (for calcification analysis only). Aβ_40_ measurements were adjusted for Aβ_42_, and Aβ_42_ for Aβ_40_. Model II: model *I* + smoking habits, alcohol consumption, systolic and diastolic blood pressure, blood pressure lowering medication, diabetes mellitus, serum total cholesterol and HDL cholesterol, lipid-lowering medication, body mass index, creatinine level, *APOE* ɛ4 carrier status. Model III: model II + mutual adjustment for AD biomarkers and calcification.

When stratifying on age, associations of arterial calcification with cognition were most profound prior age 70, attenuating thereafter (Supplementary Table 1). Associations of plasma Aβ with cognition did not differ substantially by age, except for somewhat stronger yet non-significant risk estimates in the protective direction in the oldest old (Supplementary Table 1).

### Interaction between Aβ and calcification on cognition

**Fig. 2:**
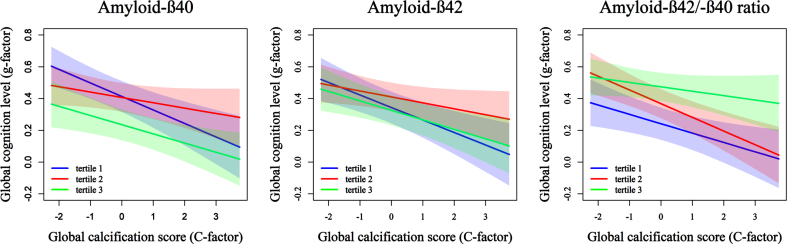
Association of systemic calcification with global cognition, stratified by tertile of plasma levels of Aβ_40_, Aβ_42_, and the Aβ_42/40_ ratio. The figure depicts the association of systemic arterial calcification level (C-factor) and the cognition outcome (g-factor) per tertile of plasma Aβ_40_, Aβ_42_, and the Aβ_42/40_ ratio.

We observed a significant interaction between Aβ_42_ and calcification on global cognition in age- and sex-adjusted models (Table 3; p_interaction_ = 0.03), such that the associations of amyloid with cognition were more profound with higher levels of arterial calcification (Fig. 2). This interaction on global cognition was also present in the Aβ_42/40_ ratio (Table 3; p_interaction_ = 0.02). The associations attenuated, and interaction terms were no longer statistically significant after adjustment for cardio metabolic risk factors (Table 3). The interaction effects between Aβ_42_ and the C-factor were most profound for motor function, followed by the memory and information processing domains (Supplementary Table 2). In terms of location of arteriosclerosis, similar patterns were observed across sites, but with strongest evidence of interaction in the presence of coronary artery calcification, followed by extracranial and intracranial carotid artery calcification (Supplementary Table 3).

**Table 3:** Association and interaction of plasma biomarkers (Aβ_40_, Aβ_42_) and systemic arterial calcification (C-factor) on global cognition outcome (g-factor)

	Global cognition (g-factor)	
	Adjusted mean difference (95% Confidence Interval)	
	Model I	Model II
**A**β_**40**_
Aβ_40_	–0.03 (–0.07; 0.02)	–0.01 (–0.06; 0.05)
Arterial calcification (C-factor)	–0.06 (–0.08; –0.04)^**^	–0.04 (–0.07; –0.01)^**^
Aβ_40_^*^ C-factor	0.01 (–0.02; 0.03)	–0.004 (–0.03; 0.02)
**A**β_**42**_
Aβ_42_	0.05 (–0.003; 0.10)	0.01 (–0.04; 0.07)
Arterial calcification (C-factor)	–0.06 (–0.08; –0.04)^**^	–0.04 (–0.07; –0.01)^**^
Aβ_42_^*^ C-factor	0.03 (0.003; 0.05)^*^	0.01 (–0.01; 0.04)
**A**β_**42/40**_ ratio
Aβ_42/40_ ratio	0.02 (–0.02; 0.06)	–0.01 (–0.05; 0.04)
Arterial calcification (C-factor)	–0.06 (–0.08; –0.04)^**^	–0.04 (–0.07; –0.01)^**^
Aβ_42/40_ ratio ^*^ C-factor	0.03 (0.001; 0.05)^*^	0.02 (–0.01; 0.04)

^*^p < 0.05; ^**^p < 0.01. Model I: adjusted for age, age^2^, sex, education, and plasma analysis batch. Mutual adjustment for AD biomarkers. Model II: model *I* + smoking habits, alcohol consumption, systolic and diastolic blood pressure, blood pressure lowering medication, diabetes mellitus, serum total cholesterol and HDL cholesterol, lipid-lowering medication, body mass index, creatinine level, *APOE* ɛ4 carrier status.

## DISCUSSION

In this cross-sectional population-based study, we found that arterial calcification and plasma AD biomarkers display additive effects on cognition in the general population. Plasma Aβ was associated with worse cognition particularly in the presence of arterial calcification. These interactive patterns were explained in part by differences in cardiovascular risk profiles.

Our findings underline that in many individuals in the general population, AD and vascular disease contribute jointly to cognitive decline, through either separate or intertwined pathways. Previous study has shown a similar interaction of neurodegenerative markers on MRI [27]. Others have hinted at a vascular contribution to the pathogenesis of AD, reporting higher burden of Aβ on PET imaging in participants with hypertension and diabetes [28, 29]. Decline in cerebral blood flow later in life may be a mediating factor between vascular pathology and Aβ accumulation [30]. Evidence has shown that impairments in blood flow occur before the more common neurological biomarkers associated with AD [31, 32]. Driven by observations that arterial calcification contributes to AD risk [33], the “vascular hypothesis of AD” was coined, stating that impaired function of the neurovascular unit affects neuronal health and cognitive ability before the classical AD biomarkers are affected and can promote the accumulation of Aβ in the brain [34–36]. Similarly, vascular dysregulation and stiffening may affect clearance of Aβ from the brain [15, 32]. However, the role of arteriosclerosis in the etiology of AD remains uncertain [37], and associations of cardiovascular risk factors with cerebral amyloid deposition have not been consistent across cohort studies [38, 39]. The attenuation of interactions after adjustment for cardiovascular risk factors in the present study suggests a role at least in both AD and vascular cognitive impairment and decline that warrants further exploration in longitudinal studies capturing change in cognitive ability over time.

The co-occurrence of Aβ and arterial calcification steeply increased with age, in line with high prevalence of different pathologies in post-mortem studies of both cognitively healthy and demented individuals [40]. In the Religious Orders Study and the Memory and Aging Project, autopsy showed that >94% of participants had at least one neuropathology at death, with the most common neuropathology being AD (65.3%) although it very rarely occurred in isolation (9%), often AD was comorbid with at least one other neurodegenerative and vascular pathology (44%) or with at least one vascular pathology (40%) [2]. While the lack of established cut-offs for plasma Aβ to determine cerebral amyloid burden render the percentages in our study a poor men’s alternative to postmortem study, they do illustrate that findings from postmortem study translate into *in vivo* effects on cognition. The high co-occurrence of AD and vascular pathology may be reflected particularly by impairment on the memory and executive domains.

Our results suggest that measurement of both arterial calcification and Aβ in the diagnostic work-up could provide additional insight in the etiology of cognitive decline in the majority of older patients with combined pathologies, above and beyond measurement of a single disease marker. Simultaneous measurement could help to determine the most likely cause for cognitive decline in patients and personalize treatment. While we operationalized the vascular component as a joint measure of arteriosclerosis across four different vessel beds, we have previously shown that a more pragmatic approach across two vessels maybe just as reliable to map the arteriosclerotic burden [25]. Further studies are needed to determine whether similar interactions are observed for cerebral small-vessel disease, and to which extent these are related to vascular stiffening due to arterial calcification. This may also clarify the role of cardio metabolic risk factors in the interplay between amyloid and vascular pathology.

Strengths of this study include the use of the high sensitivity Simoa measurements in the setting of a large population-based sample. There are limitations also. First, although recent studies have shown good validity of plasma biomarkers of AD for detecting amyloid pathology in the brain and cerebral spinal fluid, their specificity remains somewhat lower and may be affected by comorbid pathology. In the absence of an absolute measure to determine presence of amyloid in the central nervous system, we relied on the lower quartiles of Aβ (and arteriosclerosis) to demonstrate to increasing prevalence of multipathology with advancing age. Second, CT-imaging was done on average 6 months prior to measurement of cognition and plasma markers, which may have introduced some measurement error. Third, the cross-sectional design hampers inference about the interplay of the different markers in the trajectory of cognitive decline. Fourth, the vast majority of participants (>96%) were of European ancestry, rendering results potentially less generalizable to other ancestries.

In conclusion, arteriosclerosis and Aβ display additive interaction-effects on cognitive performance in the general population, that are due in part to cardio metabolic risk factors. These findings suggest that joint assessment of vascular and AD pathology is crucial for understanding disease etiology in individuals with cognitive impairment.

## Supplementary Material

Supplementary Material

## Data Availability

Anonymized data are available on reasonable request. Requests for access to the data reported in this paper can be directed to data manager Frank J.A. van Rooij (secretariat.epi@erasmusmc.nl).
