# Supplementary material for: Interaction Between Arteriosclerosis and Amyloid-β on Cognitive Function
Source: J Alzheimers Dis. 2024 Jan 16;97(2):953–61. doi: 10.3233/JAD-230604 (PMC10836547; doi:10.3233/JAD-230604)

Supplementary Material

Interaction Between Arteriosclerosis and Amyloid-β on Cognitive Function

Supplementary Table 1. Association of plasma biomarkers ($A\beta_{40}$, $A\beta_{42}$) and systemic arterial calcification (C-factor) on global cognition outcome (g-factor), stratified by age <70, 70-79, and 80+.

	Global cognition (g-factor)		
	Adjusted mean difference (95% Confidence Interval)		
	**Model I**	**Model II**	**Model III**
**Age group <70**			
$A\beta_{40}$	-0.02 (-0.08; 0.03)	-0.01 (-0.07; 0.06)	-0.003 (-0.07; 0.06)
$A\beta_{42}$	0.02 (-0.04; 0.08)	0.01 (-0.05; 0.08)	0.01 (-0.05; 0.07)
$A\beta_{42/40}$ ratio	0.01 (-0.05; 0.06)	-0.01 (-0.07; 0.05)	-0.01 (-0.07; 0.05)
Arterial calcification (C-factor)	-0.06 (-0.09; -0.03)**	-0.06 (-0.09; -0.02)**	-0.06 (-0.09; -0.02)**
**Age group 70-79**			
$A\beta_{40}$	-0.09 (-0.19; 0.002)	-0.01 (-0.12; 0.10)	0.004 (-0.10; 0.11)
$A\beta_{42}$	0.15 (0.05; 0.26)**	0.03 (-0.08; 0.14)	0.01 (-0.10; 0.12)
$A\beta_{42/40}$ ratio	0.09 (0.01; 0.18)*	0.01 (-0.08; 0.10)	-0.01 (-0.10; 0.08)
Arterial calcification (C-factor)	-0.05 (-0.10; -0.01)*	-0.01 (-0.06; 0.04)	-0.01 (-0.06; 0.04)
**Age group 80+**			
$A\beta_{40}$	0.03 (-0.16; 0.21)	0.08 (-0.23; 0.39)	0.08 (-0.24; 0.41)
$A\beta_{42}$	0.01 (-0.19; 0.21)	-0.15 (-0.49; 0.18)	-0.16 (-0.52; 0.19)
$A\beta_{42/40}$ ratio	-0.01 (-0.17; 0.14)	-0.15 (-0.43; 0.13)	-0.16 (-0.45; 0.14)
Arterial calcification (C-factor)	-0.14 (-0.24; -0.03)*	0.02 (-0.15; 0.19)	0.01 (-0.17; 0.19)

*p<0.05; **p<0.01

Model I: adjusted for age, age^2, sex, education, batch number (for plasma analysis only), and scanner used and time interval between scanning and cognitive assessment (for calcification analysis only). $A\beta_{40}$ measurements were adjusted for $A\beta_{42}$, and $A\beta_{42}$ for $A\beta_{40}$.

Model II: model I + smoking habits, alcohol consumption, systolic and diastolic blood pressure, blood pressure lowering medication, diabetes mellitus, serum total cholesterol and HDL cholesterol, lipid-lowering medication, body mass index, creatinine level, *APOE* ε4 carrier status.

Model III: model II + mutual adjustment for AD biomarkers and calcification.

Supplementary Table 2. Interaction of systemic arterial calcification (C-factor) and plasma Aβ on cognitive domains.

	**Memory domain**		
	Adjusted mean difference (95% Confidence Interval)		
	$A\beta_{40}$	$A\beta_{42}$	$A\beta_{42/40}$ ratio
Aβ	-0.004 (-0.07; 0.06)	-0.003 (-0.07; 0.06)	-0.03 (-0.08; 0.03)
Calcification (C-factor)	-0.003 (-0.04; 0.03)	-0.002 (-0.04; 0.03)	-0.003 (-0.04; 0.03)
Aβ * calcification	-0.02 (-0.05; 0.02)	-0.01 (-0.04; 0.02)	0.001 (-0.03; 0.03)
	**Executive domain**		
	Adjusted mean difference (95% Confidence Interval)		
	$A\beta_{40}$	$A\beta_{42}$	$A\beta_{42/40}$ ratio
Aβ	-0.004 (-0.05; 0.04)	0.02 (-0.03; 0.04)	0.004 (-0.04; 0.04)
Calcification (C-factor)	-0.04 (-0.06; -0.02)**	-0.04 (-0.06; -0.02)**	-0.04 (-0.06; -0.02)**
Aβ * calcification	-0.01 (-0.03; 0.01)	-0.001 (-0.02; 0.02)	0.01 (-0.02; 0.03)
	**Information processing**		
	Adjusted mean difference (95% Confidence Interval)		
	$A\beta_{40}$	$A\beta_{42}$	$A\beta_{42/40}$ ratio
Aβ	-0.001 (-0.06; 0.04)	0.03 (-0.02; 0.09)	0.02 (-0.03; 0.07)
Calcification (C-factor)	-0.04 (-0.07; -0.02)**	-0.04 (-0.07; -0.02)**	-0.04 (-0.07; -0.02)**
Aβ * calcification	0.01 (-0.02; 0.03)	0.001 (-0.02; 0.03)	-0.002 (-0.03; 0.02)
	**Motor function**		
	Adjusted mean difference (95% Confidence Interval)		
	$A\beta_{40}$	$A\beta_{42}$	$A\beta_{42/40}$ ratio
Aβ	0.01 (-0.05; 0.07)	-0.02 (-0.08; 0.07)	-0.01 (-0.06; 0.04)
Calcification (C-factor)	-0.05 (-0.08; -0.02)**	-0.05 (-0.08; -0.02)**	-0.05 (-0.08; -0.02)**
Aβ * calcification	0.03 (-0.002; 0.06)	0.05 (0.02; 0.07)**	0.04 (0.01; 0.06)*

*p<0.05; **p<0.01. Estimates of amyloid and calcification are presented per 1-SD increase. e.g., estimates in the column '$A\beta_{40}$' represent the effect estimates for $A\beta_{40}$, the C-factor, and the $A\beta_{40}$*C-factor interaction term. All estimates are from the fully adjusted model II, including age, age^2, sex, education, plasma analysis batch, interval between scanning and cognitive assessment, smoking habits, alcohol consumption, systolic and diastolic blood pressure, blood pressure lowering medication, diabetes mellitus, serum total cholesterol and HDL cholesterol, lipid-lowering medication, body mass index, creatinine level, *APOE* ε4 carrier status. Mutual adjustment for AD biomarkers.

Supplementary Table 3. Interaction of arterial calcification and plasma Aβ on global cognition outcome (g-factor).

	**Global cognition (g-factor)**		
	Adjusted mean difference (95% Confidence Interval)		
	$A\beta_{40}$	$A\beta_{42}$	$A\beta_{42/40}$ ratio
**Coronary artery**			
Aβ	0.03 (-0.07; 0.13)	-0.03 (-0.14; 0.05)	-0.09 (-0.19; 0.004)
Calcification (presence)	-0.02 (-0.11; 0.08)	-0.002 (-0.09; 0.09)	-0.02 (-0.11; 0.08)
Aβ * calcification	-0.05 (-0.15; 0.05)	0.06 (-0.05; 0.16)	0.11 (0.01; 0.21)*
**Aortic Arch**			
Aβ	-0.03 (-0.19; 0.12)	0.01 (-0.13; 0.15)	0.02 (-0.11; 0.16)
Calcification (presence)	-0.05 (-0.17; 0.08)	-0.05 (-0.17; 0.08)	-0.05 (-0.17; 0.08)
Aβ * calcification	0.03 (-0.13; 0.19)	0.01 (-0.14; 0.15)	-0.03 (-0.17; 0.11)
**Extracranial carotid artery**			
Aβ	-0.03 (-0.11; 0.06)	-0.04 (-0.13; 0.05)	-0.05 (-0.16; 0.04)
Calcification (presence)	-0.04 (-0.12; 0.04)	-0.04 (-0.12; 0.04)	-0.05 (-0.13; 0.03)
Aβ * calcification	0.02 (-0.07; 0.11)	0.07 (-0.02; 0.16)	0.05 (-0.04; 0.15)
**Intracranial carotid artery**			
Aβ	0.01 (-0.09; 0.11)	-0.01 (-0.11; 0.09)	-0.05 (-0.14; 0.04)
Calcification (presence)	-0.02 (-0.11; 0.08)	-0.01 (-0.10; 0.09)	-0.02 (-0.11; 0.07)
Aβ * calcification	-0.02 (-0.12; 0.09)	0.03 (-0.07; 0.13)	0.06 (-0.03; 0.16)

*$p<0.05$; **$p<0.01$. Estimates of amyloid are presented per 1-SD increase, calcification is presented as presence of calcification. e.g., estimates in the 'coronary artery' related rows and column '$A\beta_{40}$' represent the effect estimates for $A\beta_{40}$, presence of coronary calcification, and the $A\beta_{40}$*coronary calcification interaction term. All estimates are from the fully adjusted model II, including age, age^2, sex, education, plasma analysis batch, interval between scanning and cognitive assessment, smoking habits, alcohol consumption, systolic and diastolic blood pressure, blood pressure lowering medication, diabetes mellitus, serum total cholesterol and HDL cholesterol, lipid-lowering medication, body mass index, creatinine level, *APOE* ε4 carrier status. Mutual adjustment for AD biomarkers.

Supplementary Figure 1. CT image of calcification in vessel beds. Example of calcification in examined vessel beds (left: coronary artery, middle: aortic arch, right: carotid artery bifurcation). Top row shows calcification, bottom row depicts the segmentation of the calcification. Calcification was quantified by calculation of the Agatston score for each lesion.

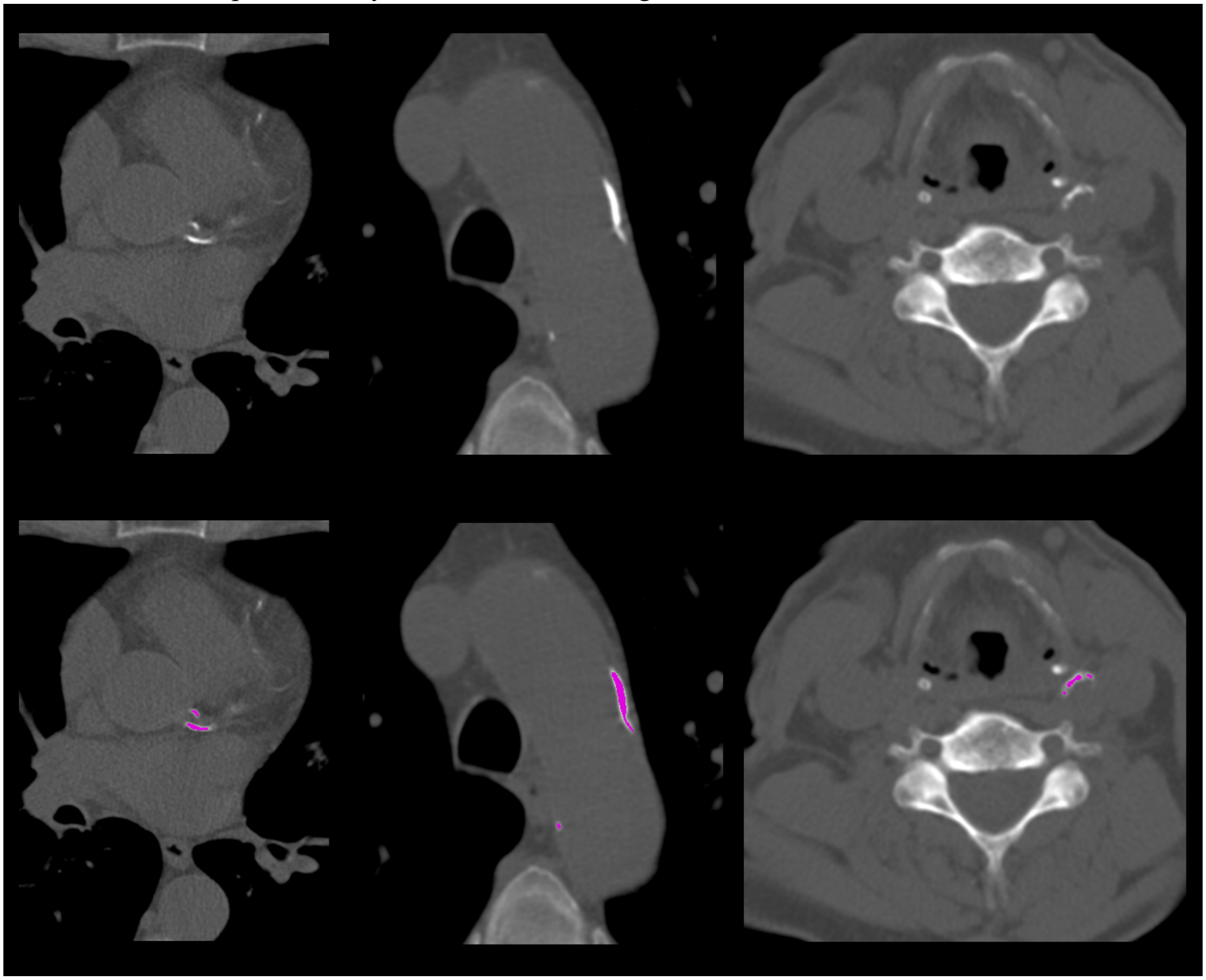

Supplementary Figure 2. Correlation matrix of plasma amyloid and C-factor with the risk factor covariates. Correlation matrix of plasma amyloid and C-factor with cardiovascular risk factor covariates used in the regression models. Plasma Aβ_{40} was correlated with plasma Aβ_{42}. A correlation of age with plasma Aβ_{40} and C-factor can be observed. Systolic and diastolic blood pressure were correlated.

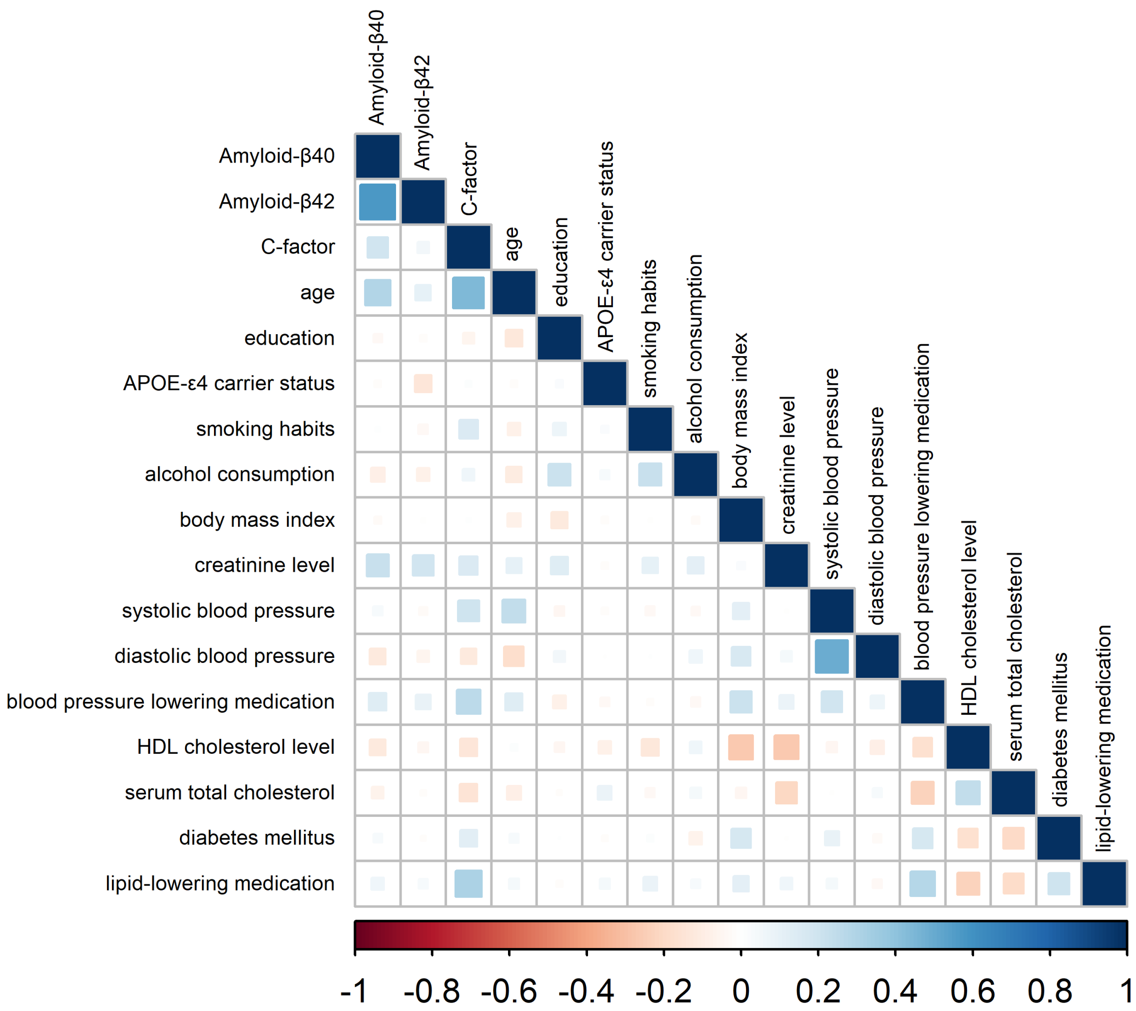

Supplement: Supplementary Material [file jad-97-jad230604-s001.pdf]
